# Fellow-Workers and the Roll of Honour

**Published:** 1914-10-31

**Authors:** 


### ROUND THE WORLD.

[The Editor Invites Early Information and Contributions Marked "Fellow-Workers."]

### Fellow-Workers and the Roll of Honour.

The following members of the staff of the Central London Throat, Nose, and Ear Hospital, Gray's Inn Road, W.C., are giving their services to the nation just now: Mr. George Potts, F.R.C.S. Edin.; Mr. Colin McKenzie, F.R.C.S. Eng.; Dr. Adam Gray, M.D.; Dr. Nicol M. Rankin, M.B.; and Mr. Leslie Rawes, M.R.C.S., L.R.C.P., the first four being registrars, and the last-named anæsthetist, to the institution. Mr. George Potts is a resident of Maidstone, where he holds the appointment of honorary aural surgeon to the Kent County Ophthalmic Hospital, to which he was at one time house-surgeon. He mobilised as captain in the 1st Home Counties Field Ambulance of the Territorial R.A.M.C.

Mr. Colin McKenzie is a Cambridge and Middlesex man, and won the junior Broderip scholarship at the last-mentioned institution in 1908. Recently he was resident surgical officer to the Royal Infirmary at Bradford, whilst Dr. Adam Gray is an M.D., Ch.B. Aberd., and has lately held the appointments of clinical assistant in the throat department of the Brompton Hospital for Consumption and house-surgeon to the Oldham Infirmary.

Mr. Leslie Rawes mobilised as lieutenant in the 2nd City of London Field Ambulance. A St. Thomas's man, he has held several important posts since qualification in 1905, including those of house-surgeon to the Great Northern Central Hospital and resident medical officer to the Royal Waterloo Hospital for Children and Women.

Mr. Percy Sargent, F.R.C.S., who has taken charge of the surgical division of the British Red Cross Hospital in Rouen, is a well-known member of the staff of St. Thomas's Hospital, where he holds the post of surgeon to out-patients. After spending his first years as a medical student at Cambridge he gained a University entrance scholarship at St. Thomas's in 1895, and became an F.R.C.S. five years later. Mr. Sargent is also surgeon to the National Hospital for Paralysis and Epilepsy. He was an Erasmus Wilson lecturer at the Royal College of Surgeons in 1905.

Mr. Frederic Francis Burghard, who has left for the Front as a consulting surgeon to the Expeditionary Force, has been surgeon to King's College Hospital since 1897. A student of "Guy's," where he had a distinguished academic career, Mr. Burghard became an M.D., M.S., and F.R.C.S. Eng. Then, having fulfilled the appointment of surgical registrar at "Guy's," he became assistant surgeon to King's College Hospital in 1890, where he quickly made a name for himself both as a teacher and as an operator. Among other important appointments he holds the posts of senior surgeon to the Paddington Green Children's Hospital and examiner in surgery to the Universities of London and Cambridge. Mr. Burghard has a country house at Hildenborough, in Kent, where he is much interested in horticulture.

Dr. Thomas Renton Elliott, M.D., M.R.C.P., who is accompanying Sir John Rose Bradford to the Continent, is a brilliant member of the junior staff at University College Hospital, where he is an assistant physician. As a student Dr. Elliott carried off many important prizes, including more than one gold medal, and from his earliest days at Cambridge evinced an aptitude for research which in later years brought him the coveted distinction of F.R.S. Much of Dr. Elliott's research work has been in connection with the sympathetic nervous system and the ductless glands.

Mr. Alexander Fleming, M.B., B.S. Lond., F.R.C.S. Eng., who is accompanying Sir Almroth Wright to the Front, is a St. Mary's man who early in his career took up work in the inoculation department of his hospital and quickly achieved distinction for his researches. Mr. Fleming won several scholarships as a student, and at his final London University examination was awarded a gold medal with honours in medicine, pathology, forensic medicine, and hygiene. Some of his more important work has been in connection with the Wassermann reaction and the salvarsan treatment.

Many distinguished physicians and surgeons from Aberdeen, Edinburgh, and Glasgow mobilised to form the four Scottish general hospitals of the Territorial establishment. The first of these is at Aberdeen, in command of Lieutenant-Colonel P. Mitchell, who has under him Dr. A. M. Booth, Dr. H. A. Lister, Dr. A. W. Macintosh, and Dr. J. Marnoch, who hold the rank of lieutenant-colonels.

Dr. James Mackenzie Booth has been in practice for many years at Aberdeen, where he qualified M.B., C.M., in 1877, subsequently becoming an M.D. of his University. Well known as a throat and ear surgeon, Dr. Booth, who was formerly chloroformist to the Aberdeen Royal Infirmary, is now consulting surgeon to that institution. He is also lecturer on diseases of the ear and larynx to the University, and surgeon to the throat and ear department of the Aberdeen General Dispensary.

Dr. Arthur Hugh Lister, M.D. Aberd., is physician and lecturer on clinical medicine to the Aberdeen Royal Infirmary. He studied at King's College Hospital as well as at Aberdeen, and has held numerous important appointments, including those of honorary physician to the tuberculosis ward of the Aberdeen City Hospital, and medical officer to the Morningfield Hospital for Incurables there.

Dr. Ashley Watson Macintosh is a well-known Aberdeen physician and a prominent member of the Royal Infirmary staff. He has had a distinguished academic career, and gained highest honours when graduating as a doctor of Aberdeen University, where he now holds the important post of Regius professor of medicine. Dr. Macintosh, who was in earlier years a student of King's College Hospital, and clinical assistant to the National Hospital for Epilepsy and Paralysis, in Queen Square, has written largely on diseases of the brain and spinal cord.

Mr. John Marnoch, M.B., C.M. Aberd., occupies amongst the surgeons of Aberdeen a position similar to that enjoyed on the medical side by Dr. Macintosh. Formerly assistant to the professor of physiology at Aberdeen, he is now Regius professor of surgery there as well as surgeon and lecturer on clinical surgery to the Royal Infirmary.

